# Specific, Sensitive, and Quantitative Enzyme-Linked Immunosorbent Assay for Human Immunoglobulin G Antibodies to Anthrax Toxin Protective Antigen

**DOI:** 10.3201/eid0810.020380

**Published:** 2002-10

**Authors:** Conrad P. Quinn, Vera A. Semenova, Cheryl M. Elie, Sandra Romero-Steiner, Carolyn Greene, Han Li, Karen Stamey, Evelene Steward-Clark, Daniel S. Schmidt, Elizabeth Mothershed, Janet Pruckler, Stephanie Schwartz, Robert F. Benson, Leta O. Helsel, Patricia F. Holder, Scott E. Johnson, Molly Kellum, Trudy Messmer, W. Lanier Thacker, Lilah Besser, Brian D. Plikaytis, Thomas H. Taylor, Alison E. Freeman, Kelly J. Wallace, Peter Dull, Jim Sejvar, Erica Bruce, Rosa Moreno, Anne Schuchat, Jairam R. Lingappa, Sandra K. Martin, John Walls, Melinda Bronsdon, George M. Carlone, Mary Bajani-Ari, David A. Ashford, David S. Stephens, Bradley A. Perkins

**Affiliations:** * Centers for Disease Control and Prevention, Atlanta, Georgia, USA; † Emory University, Atlanta, Georgia, USA

**Keywords:** *Bacillus anthracis*, anthrax, antibody, assay, toxin, bioterrorism, ELISA, serology

## Abstract

The bioterrorism-associated human anthrax epidemic in the fall of 2001 highlighted the need for a sensitive, reproducible, and specific laboratory test for the confirmatory diagnosis of human anthrax. The Centers for Disease Control and Prevention developed, optimized, and rapidly qualified an enzyme-linked immunosorbent assay (ELISA) for immunoglobulin G (IgG) antibodies to *Bacillus anthracis* protective antigen (PA) in human serum. The qualified ELISA had a minimum detection limit of 0.06 µg/mL, a reliable lower limit of detection of 0.09 µg/mL, and a lower limit of quantification in undiluted serum specimens of 3.0 µg/mL anti-PA IgG. The diagnostic sensitivity of the assay was 97.8%, and the diagnostic specificity was 94.2%. A competitive inhibition anti-PA IgG ELISA was also developed to enhance diagnostic specificity to 100%. The anti-PA ELISAs proved valuable for the confirmation of cases of cutaneous and inhalational anthrax and evaluation of patients in whom the diagnosis of anthrax was being considered.

Naturally occurring anthrax is a zoonotic disease of herbivores, with low-level sporadic infection of humans. Since 1950, human anthrax in the United States was confined to those occupationally at risk, with only 235 confirmed cases, mostly cutaneous, reported from 1955 to 2002 (1–3). The occurrence of human anthrax in the country and the public perception of the disease changed dramatically in the fall of 2001, with the first successful bioterrorist anthrax attack on the U.S. civilian population. This event necessitated the simultaneous development and application of qualified laboratory assays—including serologic assays—to evaluate patients suspected of having anthrax.

The major obstacle to serologic analysis of human anthrax has been the lack of assay standardization. Variations in antigen preparation and purity, assay methods, and endpoint determination between laboratories and the absence of a suitable standard reference serum compound this problem. The Centers for Disease Control and Prevention (CDC) had, before the attacks, instituted the development of anthrax serologic assays—particularly enzyme-linked immunosorbent assays (ELISAs)—for use in anthrax vaccine studies in humans and to provide a standard human reference serum. In response to the anthrax emergency of 2001, we report the accelerated development and qualification of a quantitative ELISA for detection of anti-protective antigen (PA) specific immunoglobulin (Ig) G in human serum and the development of a competitive inhibition assay to enhance diagnostic specificity. The assays were applied to diagnosis of cutaneous and inhalational anthrax to evaluate serologic responses in persons considered at risk from anthrax spore exposure and enhance anthrax serologic tests with standardized techniques for distribution to public health and clinical laboratories.

## Methods

### Antigen Preparation

Recombinant anthrax toxin protective antigen (rPA) with an amino acid sequence concurring with that from the *Bacillus anthracis* V770-NP1-R anthrax vaccine strain was obtained from the National Institute of Craniofacial and Dental Research, National Institutes of Health, Bethesda, MD. Antigen was stored frozen at –80°C in small aliquots (10–100 µL, 4.75 mg/mL) in 5 mM Hepes, pH 7.3. Antigen was expressed from the attenuated asporogenous host *B. anthracis* BH445 and purified to homogeneity as described (4).

### Human Serum for Determination of Diagnostic Specificity and Sensitivity

To determine the background level of anti-PA ELISA reactivity in a cross-section of the U.S. population, a panel of 238 control sera from healthy adult persons was assembled from the CDC Occupation Health Service and the National Health and Nutrition Examination Survey (NHANES, CDC) serum collections. Donors were selected on the basis of having no known exposure to *B. anthracis* or anthrax and no known history of anthrax vaccination. In addition, a panel of 277 sera was assembled from persons with clinically confirmed non-anthrax-related illnesses (acute hepatitis A, acute hepatitis B, influenza A and B, brucellosis, staphylococcal toxic-shock syndrome, group A streptococcal infections, legionellosis, *Chlamydia pneumoniae* infection, and *Mycoplasma pneumoniae* infection) and from children and adults who had received non-anthrax-related vaccines (trivalent influenza, hepatitis B, tetanus toxoid, and botulinum toxoid). To determine assay sensitivity, an additional panel of 68 sera from persons who had received anthrax vaccine adsorbed (AVA) and 19 control sera from nonvaccinees was obtained. All sera were tested in duplicate without heat inactivation.

### Human Standard Serum Preparation

The anti-AVA standard human reference serum, AVR414, was prepared by plasmapheresis of healthy adult CDC volunteers who had received at least four subcutaneous injections of Anthrax Vaccine Adsorbed (AVA, BioPort Corp., Lansing, MI) with the licensed regimen (0, 2, and 4 weeks; 6, 12, and 18 months; and yearly boosters). Plasmapheresis and serum conversion were done at the Emory Transfusion Medicine Program, Emory University School of Medicine (Atlanta, GA) and the Scientific Resource Program at CDC, respectively. Plasmapheresis was done by the TPE DUAL- NEEDLE procedure with the COBE Spectra Apheresis System (Gambro BCT, Inc., Blood Component Technology, Lakewood, CO) and following the manufacturer’s procedure manual (Manual #701900–000 1999/1). Each plasma unit was clotted with sterile glass microbeads (B. Braun Instruments, Burlingame, CA) and suspended in 1.5 M CaCl_2_–2.0 M ε-amino-caproic acid. All units were allowed to clot overnight at room temperature and were then centrifuged at 2,200 x *g* at 4°C for 15 min. The serum from each unit was stored in a 500-mL sterile plastic container. The level of residual anticoagulants was not measured. The total IgG concentration of the serum pool was determined by radial immunodiffusion and nephelometry, with the U.S. National Reference Preparation for Specific Human Serum Proteins (CDC) as a standard (5). Anti-PA specific IgG mass value assignment to the standard serum was done by differential adsorption, homologous enzyme-linked immunoassay (EIA), and heterologous ELISA (Semenova VA, et al., manuscript in preparation), with U.S. Food and Drug Administration (FDA) 1983 *Haemophilus influenzae* type b (Hib) reference serum (6).

### ELISA Procedure

Polyoxyethylene sorbitol monolaurate (Tween 20) was purchased from BioRad Laboratories (Hercules, CA). Skim milk powder was obtained from Difco/Becton Dickinson (Atlanta, GA). Horseradish peroxidase (HRPO)–conjugated mouse anti-human IgG (affinity purified, γ-chain specific monoclonal clone HP6043) was obtained from Hybridoma Reagent Laboratories (Baldwin, MD). Peroxidase substrate 2,2´-azino-di(3-ethyl-benzthiazoline-6-sulfonate) (ABTS), hydrogen peroxide (H_2_O_2_), and peroxidase stop solution were obtained from Kirkegaard & Perry Laboratories (KPL, Gaithersburg, MD). All other laboratory reagents were obtained from Sigma Chemical Co. (St. Louis, MO) unless otherwise specified. Sterile, Type I endotoxin-free water was used for all ELISA procedures.

Immulon II-HB flat-bottom 96-well microtiter plates (Thermo Labsystems, Franklin, MA), were coated for 16 hrs at +4°C with 100 µL/well of rPA at a concentration of 2.0 µg/mL in 0.01 M phosphate-buffered saline (PBS), pH 7.4 (Life Technologies, Gaithersburg, MD). Plates were stored at +4°C without blocking and used within 7 days of preparation. Antigen-coated plates were then washed three times (ELX405 microplate washer, BioTek Instruments Inc., Winooski, VT) with PBS containing 0.1% Tween 20 and blotted dry by inversion on clean paper towels. Control and serum antibodies were tested without a separate blocking step. Serum standards and sera for testing were prepared at the appropriate dilutions in PBS containing 5% skim milk and 0.5% Tween 20, pH 7.4. The human standard reference serum and test sera were serially diluted twofold in the plate in the same buffer solution. The minimum dilution of test serum was 1/50. Three positive control sera from three separate donors and one negative control serum were each used at single dilution factors selected to give a range of optical density (OD) values across the standard reference curve. The final volume in all wells was 100 µL.

Test and standard sera were incubated in a humidified chamber (covered tray) for 60 min at 37°C, and the plates were then washed three times with PBS containing 0.1% Tween 20. Bound anti-PA IgG was then detected by using HRPO-conjugated mouse anti-human IgG Fc PAN monoclonal HP6043 diluted in PBS containing 5% skim milk and 0.5% Tween 20 (100 µL/well), and plates were incubated in a humidified chamber (covered tray) for 60 min at 37°C. Plates were again washed three times with PBS containing 0.1% Tween 20, and bound conjugate was detected colorimetrically by using ABTS/H_2_O_2_ substrate (100 µL/well). Color development was over 30 min (±∀5 min) and was stopped by addition of 100 µL of Peroxidase Stop Solution (KPL) to all wells of the test plates. OD values were read within 30 min of addition of the stop solution with a MRX Revelation microtiter plate reader (Thermo Labsystems, Franklin, MA) at a wavelength of 410 nm with a 610-nm reference filter. Data were analyzed by using a four-parameter (4-PL) logistic-log curve fitting model with ELISA for Windows software (7). A calibration factor for the standard reference serum was used to determine the concentration of anti-PA IgG in micrograms per milliliter of serum (µg/mL).

### Competitive Inhibition ELISA

To enhance specificity, a supplementary rPA competitive inhibition ELISA (CI-ELISA) was developed based on the qualified anti-PA IgG ELISA. The CI-ELISA was a direct extension of the standard ELISA procedure with the following modifications. The anti-PA antibody concentrations of the test sera were first determined by using the standard ELISA. Only sera with a minimum reactivity level of 10 µg/mL anti-PA antibody were suitable for evaluation in the CI-ELISA. The 10-µg/mL threshold was determined empirically as the minimum level for which a reduction in ELISA reactivity could be assigned with statistical significance. A concentration of 50 µg rPA/500 μL diluted sample was chosen as the absorbing concentration after a preliminary study with ranges between 0 and 200 µg/mL (8). Test sera were then diluted to a concentration calculated to provide an OD value of approximately 1.0, based on their reactivity in the standard anti-PA ELISA. A 1-mL volume of each diluted serum was prepared and divided into two aliquots of equal volume. To one of these aliquots, rPA was added to a final concentration of 100 µg/mL. Both tubes were capped tightly and mixed by inversion for 16–18 hrs at +4°C. After this incubation, the tubes were centrifuged at 4°C for 10 min at 8,000 x *g* to remove precipitated materials. Test sera were incubated in the presence and absence of an excess of rPA in solution before analysis in the standard ELISA.

The supernatants were used without further dilution in the standard ELISA described above. Based on defined sera from anthrax vaccine recipients and confirmed clinical cases, a ≥85% suppression of reactivity in the competitive ELISA was identified as the threshold to discriminate between true positives and false positives.

### Accuracy, Precision, Limits of Quantification, and Goodness of Fit

Accuracy describes the exactness of the assay to measure a known, true value of anti-PA IgG and to measure it repeatedly. In this study, accuracy was determined by repeated analysis of a positive control human anti-AVA antiserum for which differential absorption and heterologous ELISA had determined the anti-PA IgG concentration. Accuracy is expressed as the percent error between the assay-determined value and the assigned value for that serum. A percent error of ≤20% is an acceptable level of accuracy for an enzyme immunoassay (9). Precision, a measure of the degree of repeatability of an assay under normal operating conditions, is expressed as the coefficient of variation of the concentrations calculated for the standard reference curve dilutions within a single assay plate (intraassay precision) and between different assay plates (interassay precision) determined over time and controlling for different operators. Acceptable levels of intraassay and interassay precision are 10% and 20%, respectively (9), and these can be used to define the range of the assay and the upper and lower limits of quantification. The range of the assay is the interval between the upper and lower levels of antibody (inclusive) that have been demonstrated to be determined with these levels of precision and accuracy.

The “goodness of fit” of the assay is, for comparative purposes, an indication of how closely the data points of the reference serum standard curve fit the 4-PL model. Goodness of fit is expressed as the regression coefficient (R^2^) of the standard curve. An R^2^ value that approaches unity is indicative of a good fit for the data to the curve (9).

### Limits of Detection of the Anti-PA IgG ELISA

The 4-PL function was used to model the characteristic curve for the standards data. These data exhibit a sigmoidal shape when plotted on an OD-log_10_ dilution scale. The 4-PL function fits these data with a high degree of accuracy and extends the range of the assay, thus providing a more precise measurement of antibody concentration for patient sera (10). The lowest concentration of analyte (anti-PA IgG) that can be detected with a specific degree of probability in a diluted serum sample is defined as the minimum detectable concentration (MDC). The lowest concentration of analyte that has a high probability of producing a response significantly greater than the response at zero concentration of analyte is defined as the reliable detection limit (RDL). The MDC and RDL of the anti-PA IgG ELISA were derived from a 4-PL fit applied to the AVR414 standard reference serum (9). The MDC is the concentration of anti-PA antibody corresponding to the interpolated intersection of the lower asymptote of the upper 95% confidence interval (95% CI) with the 4-PL fit of the standards data. The RDL is the concentration of anti-PA antibody corresponding to the interpolated intersection of the upper 95% CI asymptote with the lower-95% CI of the standards data. The MDC and RDL are thus both derived from the 95% CIs of the standard curve. They are distinct and statistically robust measurements of the lower limits of detection of the assay; the RDL is the more conservative of the two. An illustration of the relationship of MDC and RDL to the standard curve is shown (Figure).

**Figure:**
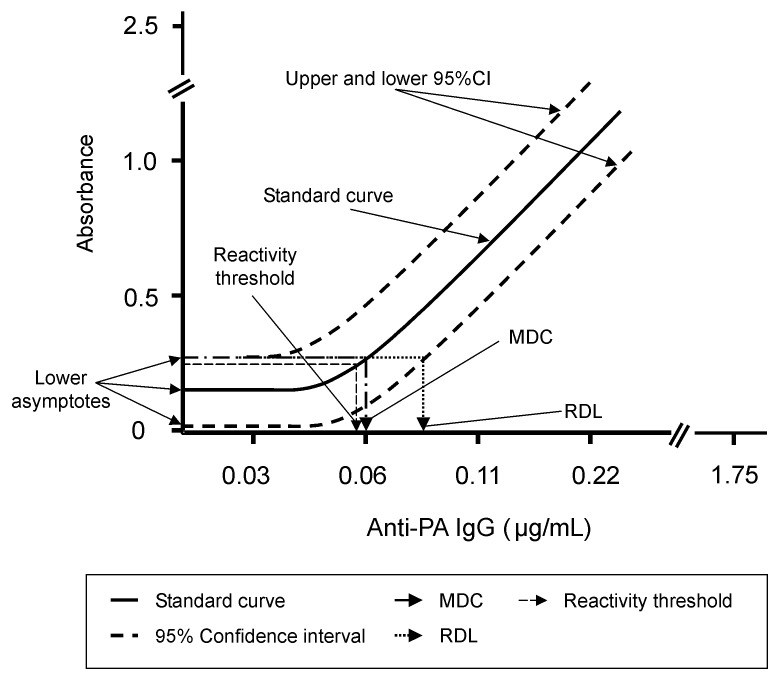
Graphic representation of minimum detectable concentration (MDC), reliable detection limit (RDL), and reactivity threshold. The MDC is the concentration of anti-protective antigen antibody (anti-PA) corresponding to the interpolated intersection of the lower asymptote of the upper 95% confidence limit of the 4-parameter logistic log fit of the standard curve data. The RDL is the concentration of anti-PA antibody corresponding to the interpolated intersection of the lower asymptote of the upper 95% confidence limit with the lower 95% confidence limit of the standard’s data. The reactivity threshold was determined as the upper 95% confidence limit of the frequency distribution from log__10__-transformed optical density (OD) values of control human sera tested at 1/50 dilution. This OD value was converted to an anti-PA immunoglobulin (Ig) G concentration by using the standard curve calibration factor. Where this calculated value is below the MDC of the assay, the MDC was selected as the default reactivity threshold.

The reactivity threshold (Figure) is used to categorize a serum as reactive or nonreactive and to determine the diagnostic sensitivity (DSN) and diagnostic specificity (DSP) of the assay. The reactivity threshold of this assay was determined from the frequency distribution (11) of log_10_-transformed OD values from a panel of sera from humans with non-anthrax-related clinical infections (554 observations) and a panel of control human sera (476 observations). The reactivity threshold was determined as the upper 95% CI of the frequency distribution from log_10_-transformed OD values of control human sera tested at 1/50 dilution. This OD value was converted to an anti-PA IgG concentration by using the standard curve calibration factor. Where this calculated value is below the MDC of the assay, the MDC becomes the default reactivity threshold. Ideally, the MDC, RDL, and reactivity threshold will all fall within the limits of quantification as defined above.

### ELISA Diagnostic Sensitivity and Specificity

The DSP and DSN of the anti-PA IgG ELISA were determined. The quantitative test results were categorized into reactive or nonreactive by application of the reactivity threshold. The DSP of the assay was calculated as [TN/(TN+FP)], where TN = true negatives and FP = false positives. The DSN of the assay was calculated as [TP/(TP+FN)], where TP = true positives and FN = false negatives. Initially, serum specimens from clinical anthrax cases were insufficient to be useful in determining the DSN of the anti-PA IgG ELISA. Thus, the DSN was calculated by using sera from a cohort of anthrax vaccine recipients who had received a minimum of four subcutaneous injections of AVA. The DSN of the assay was reevaluated at the end of the anthrax emergency, when a greater number of specimens from clinical cases had accumulated.

### Human Sera from Patients with Confirmed or Suspected Anthrax or Exposure to *B. anthracis* Spores

The qualified anti-PA ELISA was applied to sera from persons with confirmed or suspected anthrax cases and from persons exposed to *B. anthracis* spores. Blood was collected in serum separation tubes and allowed to clot; the serum was then separated from clotted cells by low-speed centrifugation. Serum was shipped to CDC with a unique identification number. All clinical serum samples were blinded to the laboratory team and tested in duplicate. All ELISA-reactive sera were tested a minimum of twice. The CI-ELISA was applied to single serum specimens with a reactivity of ≥10 µg/mL and when persons’ paired sera indicated reactivity in the absence of changing anti-PA antibody concentrations over time, a >4-fold rise over the calculated value for the acute serum or the assay reactivity threshold was applied.

Anti-PA IgG concentrations in test sera were calculated by interpolation to the standard reference calibration curve by using the ELISA for Windows Software Version 1.0 (7); anti-PA IgG concentrations were expressed in micrograms per milliliter of the original serum sample. For results to be reportable, the assay was required to meet a set of quality control acceptance criteria. For an acceptable level of precision, the mean anti-PA IgG concentrations for three separate quality control sera were required to calculate within 3 standard deviations (SDs) of their assigned mean concentrations; at least two of the mean anti-PA IgG concentrations for these sera were required to be within 2 SDs of their respective assigned mean values. Assay plates were also evaluated for parallelism between the standard curve and the test samples (12).

## Results

### Performance Characteristics of the Anti-PA IgG ELISA

Feasibility, standardization, and performance of an ELISA to detect IgG antibodies against the PA of *B. anthracis* were completed during the anthrax epidemic of fall 2001. A human serum pool (AVR203) for which the anti-PA IgG concentration had been determined empirically was used to establish the accuracy of the ELISA. The percent error between the assigned value and the assay-determined value was 6.5%, as determined from independent analyses by three individual operators. These data are indicative of an acceptable level of accuracy for this type of assay (9). The performance characteristics of the AVR414 standard curve and of three positive quality control sera selected from humans vaccinated with AVA were used to determine the precision (repeatability) of the anti-PA ELISA. The positive quality control sera were tested in duplicate at single dilutions selected to represent high, medium, and low OD regions of the reference serum standard curve. The percent error was 15.6% for quality control serum #1 (n=55 tests), 20.2% for quality control serum #2 (n=92 tests), and 12.5% for quality control serum #3 (n=93 tests); the average percent error from the three sera was 16.2%. The precision within a single assay plate, as expressed by the intraassay coefficient of variation of the AVR414 standard curve, was 8.5%, and the interassay precision was 17.0%. These values are within the accepted values of 10% and 20% for intraassay and interassay precision, respectively (11), and are indicative of a high level of precision for this type of assay (9). The goodness of fit (mean R^2^) for the AVR414 standard curve calculated over 54 runs was 0.99. On the basis of the determinations of accuracy and precision given above, the range of calculable concentrations from the AVA414 standard curve is 0.06–1.7 µg/mL of anti-PA IgG with an MDC and an RDL of 0.06 µg/mL and 0.09 µg/mL, respectively (n=54).

### Limits of Quantification and Reactivity Threshold

The limits of quantification are the lowest and highest concentrations of analyte that can be measured with a fixed degree of precision. The fixed degree of precision for this assay has been selected as a coefficient of variation (%CV) of ≤20% for the calibrated antibody concentration of the reference standard curve. For the anti-PA ELISA, the %CV for the calibrated antibody concentration is ≤20% for all of the standards data, indicating that the full extent of the AVR414 standard curve encompassing the MDC and RDL can be used in calculating an anti-PA IgG concentration for unknown sera and determining the reactivity threshold. The reactivity threshold for this assay was determined from the frequency distribution of log_10_-transformed OD values from non-anthrax-related human control sera. The geometric means for the OD values from 277 sera from humans with non-anthrax-related clinical infections (544 observations) and 238 (476 observations) human control sera were 0.059 (95% CI 0.018 to 0.253) and 0.050 (95% CI 0.021 to 0.138), respectively. The higher upper confidence limit of 0.253 was used for calculation of reactivity threshold. This corresponds to an anti-PA IgG concentration of 0.056 µg/mL, indicating that the lower asymptote of the standard curve is within the 95% CI of the control sera tested at the lowest dilution (1/50) used in this assay (Figure). Because the upper confidence limit of the control sera is less than the MDC (0.06 µg/mL) of the assay, the MDC of a 1/50 diluted serum becomes the default reactivity threshold and corresponds to an anti-PA IgG concentration of 3.0 µg/mL in an undiluted serum sample. By the same logic, a more conservative determination of reactivity threshold could be derived from the RDL (0.09 µg/mL) corresponding to a concentration of 4.5 µg/mL in an undiluted serum sample. However, using the MDC to derive the reactivity threshold maximizes the sensitivity of the assay without compromising specificity; thus, the reactivity threshold of 3.0 µg/mL was selected as the lower limit of quantification.

### DSN and DSP

The reactivity threshold was used to categorize sera as reactive or nonreactive and then to determine the DSN and DSP of the assay. The reactivity threshold was determined empirically, avoiding assumptions on the antibody response rate following exposure to anthrax toxin PA whether by vaccination or clinical infection. The lack of counts in the false-positive cells (Table) for vaccinee and clinical anthrax sera and the lack of counts in the false-negative cells for normal and non-anthrax infection sera can be qualified on both their known exposure to PA and their reactivity in the anti-PA IgG ELISA. Because only a few serum specimens from clinically positive anthrax cases were available at the start of this emergency response, the DSN of the anti-PA IgG ELISA was initially determined by using 68 sera from a cohort of sera donated by AVA vaccinees. For this sample cohort, the numbers of true positives and false negatives were 67 and 1, respectively. The DSN of the assay under these conditions is therefore 98.5% (Table). When the control sera from the same sample set of donors were used, the numbers of true negatives and false positives were 15 and 4, respectively, suggesting a diagnostic specificity of 78.9% for determining whether ELISA reactivity is due to an exposure to anthrax toxin PA (Table).

**Table:** Calculation of diagnostic sensitivity and diagnostic specificity by using cohorts of known vaccination or infection status^a^

Serum test group	AVA vaccinees	Non-Vaccinees	NHANES controls	Non-anthrax infections	Clinical anthrax sera^b^	Total sera of known infection and vaccination status
True positives^c^	67	0	0	0	15	87
True negatives^d^	0	15	228	260	0	503
False positives^e^	0	4	10	17	0	31
False negatives^f^	1	0	0	0	1	2
Total	68	19	238	277	16	618
Diagnostic specificity	n/a	78.9%	95.7%	93.8%	n/a	94.2%
Diagnostic sensitivity	98.5%	n/a	n/a	n/a	93.7%	97.6%

^a^AVA, anthrax vaccine adsorbed; NHANES, National Health and Nutrition Examination Survey; n/a, not applicable. ^b^Clinical anthrax sera were obtained from donors that met the Centers for Disease Control and Prevention case definition for confirmed cutaneous and inhalational anthrax. Patients were classified as either reactive or nonreactive. ^c^Sera are considered true positives if they were obtained from clinically confirmed anthrax cases or from donors with a documented history of anthrax vaccination. ^d^True-negative sera were selected on the basis of having no known exposure to *Bacillus anthracis* infection and no known anthrax vaccination. ^e^False-positive sera are defined as sera which reacted (>3.0 µg/mL) in the anti-protective antigen immunoglobulin G enzyme-linked immunosorbent assay but for which there is no history of clinical anthrax or anthrax vaccination. ^f^False-negative sera are defined as sera from donors who are documented as vaccine recipients or had clinically confirmed anthrax.

However, separate analyses of two further serum cohorts (sera from clinical infections other than anthrax and sera from NHANES negative controls) returned DSP values of 93.8% and 95.7%, respectively (Table). Combined analysis of all sera of known infection or known vaccination status, including sera from the 21 confirmed clinical anthrax cases, indicated an overall DSN of 97.8% and consequently a 2.2% frequency of potential false positives (Table).

## Discussion

The focus of this report is to describe the qualification and performance characteristics of an ELISA for anti-PA IgG antibodies and enhancement of its specificity by using a second-stage CI-ELISA. The application of these assays to the analysis of the antibody response following anthrax infection (Quinn CP et al., manuscript in preparation) and for serologic surveillance from clinical anthrax cases will be reported in detail elsewhere (13,14). Historically, if not identified and treated early, systemic anthrax in humans was invariably fatal. As a consequence, serologic assays have not featured prominently in the diagnosis of clinical anthrax and in some reports have been considered unreliable for early identification of the disease or for establishing a retrospective diagnosis (15). Serologic assays for anthrax have primarily been applied for the evaluation of immune responses to anthrax vaccines, in epidemiologic investigations of the disease in animals, and in confirmatory diagnosis of the various manifestations of anthrax in humans (16–18). A useful adjunct to serologic analysis of anthrax infection is the Anthraxin test (19), which elicits a localized delayed-type hypersensitivity reaction to intradermal injection of a complex uncharacterized extract from attenuated vegetative *B. anthracis* cells or edema fluid from *B. anthracis–*infected animals. Anthraxin has been reported to be very accurate for retrospective verification of anthrax in humans (20), to be applicable for up to 30 years after infection (15), and to have a qualitative positive correlation with anti-PA antibodies in the sera from human clinical anthrax cases as detected by ELISA (21). Anthraxin is not, however, approved by FDA as a diagnostic reagent in the United States.

For nearly 4 decades, anthrax serologic studies depended on the Ouchterlony agar gel diffusion test (16), which in turn replaced complement fixation tests and in vivo passive protection and neutralization tests (22). The development of an indirect (passive) microhemagglutination test (23) was the next major progression in anthrax serologic testing. Based on the agglutination by serum antibodies of sheep erythrocytes sensitized with partially purified culture supernatants containing anthrax toxin PA (Factor II) (24), this test provided greater sensitivity and speed than the agar gel diffusion technique. The microhemagglutination assay, however, was laborious to set up because antigen-coated erythrocytes had only a short shelf life, and variation in erythrocyte batches compromised reproducibility (25).

The microhemagglutination assay was replaced by the more sensitive and reproducible ELISA system, which has been applied in various formats including the immobilized antigen (direct) assay (25), an immobilized anti-PA antibody (antigen-capture) assay (26), and a competition ELISA (27), with, where reported, varying degrees of specificity and sensitivity (17,18,28). Turnbull et al. (29) described using ELISA to confirm anthrax in humans and demonstrated that recipients of the licensed AVA could be distinguished from persons with natural infections on the basis of their lack of reactivity to the anthrax toxin lethal factor protein. Sirisanthana et al. (17) in a serologic study of anthrax in northern Thailand and Harrison et al. (18) in a serologic study of anthrax in Paraguay described use of both ELISA and Western blot to retrospectively evaluate seroconversion in cutaneous and oral-oropharyngeal anthrax. In both studies, separate ELISAs were applied for the detection of anti-toxin and anti-capsule antibody responses, and Western blot was used to enhance the specificity of serologic diagnosis (17,18). Turnbull et al. (16) reported the detection of anti-PA antibodies in humans and animals in the Etosha National Park and also concluded that there is a residual antibody level in these populations in an area where the disease is endemic. However, the specificity and sensitivity of the ELISA used in that study were not reported, and the frequency of anti-PA antibodies in bacteriologically confirmed clinical cases was a maximum of 71% (16). The prevalence of true positive anti-PA antibody reactivity in the general human population therefore remains unknown, although our study of 515 non-anthrax-related control sera suggests that it is probably <7%.

The anti-PA ELISA developed and qualified at CDC before being applied in the anthrax emergency has a MDC of 0.06 µg/mL, an RDL of 0.09 µg/mL, and a reactivity threshold of 3.0 µg/mL anti-PA IgG. The reactivity threshold was adopted as the lower limit of quantification. The DSN of the assay is 97.8%, and the DSP is 94.2%. The CI-ELISA enhanced DSP to 100%. These results represent substantial improvements over the published sensitivities for anti-PA ELISAs of 72% (17) and 91.7% (18). Although Harrison et al. (18) reported a specificity of 100%, this was on a sample size of 18 controls, compared with the sample size reported here of 515 control sera (277 non-anthrax-related sera plus 238 NHANES controls). An additional important outcome of this study is the provision of a standard reference serum that can be used in a variety of serologic assays for the detection and quantification of anti-PA antibodies.

When evaluating the importance of a reactive serologic result, the prevalence of disease in the group of interest should first be considered. The assays reported in this study were primarily applied as a part of a panel of laboratory tests for the confirmation of clinical human anthrax in patients in whom the disease prevalence is expected to be high (30). The assays were also applied to serologic surveys of patients who may have been exposed to spores of *B. anthracis*, a group in which the disease prevalence may be expected to be low (13,14). For assays in which the specificity and sensitivity have been determined to be high (>90%), a reactive serum in a low-prevalence group has a much greater probability of being a false positive than it does in a high-prevalence group (31). Conversely, in the high-prevalence group nonreactive sera may not be indicative of the absence of disease. In practice, the 2.2% frequency of potential false positives reported here rarely presented a problem, and seroconversion was detectable by a >4-fold rise in anti-PA IgG concentration above the assay reactivity threshold or the acute-phase serum in all but three patients (data not shown), where paired samples with an appropriate time interval between them were available (0–7 days after symptoms for acute-phase sera and 14–28 days after symptoms for convalescent-phase sera).

The necessity for a rapid public health response meant that optimizing the number and timing of the patient serum collection was not always feasible or practicable. As a result, there was a high frequency of single (i.e.*,* unpaired) sera from cases under investigation. To provide adequate analysis of these single sera and also for paired sera that were reactive but did not demonstrate changing levels of reactivity with time, a competitive rPA inhibition ELISA (CI-ELISA) was developed based on the qualified anti-PA ELISA. The objective of the CI-ELISA was to increase the DSP of the ELISA by reducing the incidence of false positives. The CI-ELISA effectively demonstrated the specificity of the ELISA format by using sera from AVA vaccinees and clinically confirmed anthrax cases (8).

Although very little published information supports the suggestion that antibiotic therapy can suppress the humoral immune response (32), anthrax infection studies in nonhuman primates have shown that early antibiotic treatment after a known challenge with *B. anthracis* spores abrogates an anti-PA antibody response (33). A plausible explanation for this is that early intervention in the infection process minimizes antigen presentation to the immune system. The implication, particularly for cutaneous anthrax in the context of a response to a bioterrorist attack, when antibiotic intervention is likely to be rapid and aggressive (2), is that serologic tests should not be used as the sole confirmatory tests for anthrax.

## Conclusion

In this bioterrorism-related anthrax outbreak, the rapid adaptation and laboratory qualification of a quantitative serologic assay for IgG antibodies to the PA component of anthrax toxin contributed to the emergency public health response. The qualified ELISA is accurate, sensitive, specific, reproducible, and quantitative, providing fractional concentrations of anti-PA IgG antibodies. This assay, together with the supplemental CI-ELISA, proved to be an invaluable tool for assisting in early diagnosis of cutaneous and inhalational anthrax cases.

Timing of the sample and specimen quality are critical elements in successful confirmation of anthrax, particularly cutaneous anthrax, where antibiotic therapy has been implemented and the onset of antibody production may be later and of lower magnitude than for inhalational anthrax. To provide an accurate clinical picture on which to base diagnosis and thus treatment, serologic testing is most appropriately used as one of a series of laboratory tests, together with a known exposure or clinical presentation consistent with anthrax.

Ongoing studies on the ability of reactive serum from clinical cases to neutralize anthrax toxin in vitro in a macrophage cytotoxicity assay may help to better describe the complex picture of immune responses to anthrax and determine whether the detection of a serologic response to anthrax toxin PA in humans infected during the bioterrorist attack of fall 2001 indicates protection against further exposure to this disease.
